# Supplementary material for: Effects of different types and frequencies of early rehabilitation on ventilator weaning among patients in intensive care units: A systematic review and meta-analysis
Source: PLoS One. 2023 Apr 24;18(4):e0284923. doi: 10.1371/journal.pone.0284923 (PMC10124886; doi:10.1371/journal.pone.0284923)
Supplement: S1 Appendix — (DOCX) [file pone.0284923.s002.docx]

S1 Appendix. Search strategy

**The full search strategies for PubMed,** **EMBASE, the Cochrane Library, and Airiti Library of clinical trials.**

#1 ("critical illness" or "ICU" or "Recovery Room" or "Respiratory Care Units" or "ICU-AW" or "Critical Illnesses" or "Illness, Critical" or "Illnesses, Critical" or "Critically Ill")

#2 ("physical therapy" or "PT" or "Rehabilitation" or "Early mobility" or " Active movement" or "AROM exercise" or "AAROM exercise" or "high intensity " or "High frequency")

#3 ("physical therapy" or "PT" or "Rehabilitation" or "PROM exercise" or "FES" or "low intensity" or "low frequency" or “usual care)

#4 ("Weaning, Ventilator" or "Respirator Weaning" or "Weaning, Respirator" or "Mechanical Ventilator Weaning" or "Ventilator Weaning, Mechanical" or "Weaning, Mechanical Ventilator" or "Airway Extubation" or "Extubation, Endotracheal" or "Tracheal Extubation")

#5 #1 AND #2

#6 #1 AND #3

#7 #1 AND #4

#8 #2 AND #3

#9 #2 AND #4

#10 #3 AND #4

#11 #1 AND #2 AND #3

#12 #1 AND #2 AND #4

#13 #1 AND #3 AND #4

#14 #2 AND #3 AND #4

#15 #1 AND #2 AND #3 AND #4 Filter: Randomized Controlled Trail

Articles were searched until 2021/12/31.

Article search was performed in January 2022.

**Database Search Histories**

| No | Search ID# | PubMed | Embase | Cochrane | Airiti |
| --- | --- | --- | --- | --- | --- |
| 1 | # 1 | 9,136 | 26,682 | 13,111 | 96,153 |
| 2 | # 2 | 95,343 | 106,783 | 44,390 | 9,593 |
| 3 | # 3 | 1,418 | 113,148 | 49,207 |  |
| 4 | # 4 | 82,991 | 956 | 396 | 5,588 |
| 5 | #1 AND #2 | 1,371 | 1,523 | 688 | 83 |
| 6 | #1 AND #3 | 1,279 | 1,592 | 781 |  |
| 7 | #1 AND #4 | 422 | 197 | 91 | 58 |
| 8 | #2 AND #3 | 68,329 | 102,870 | 42,856 |  |
| 9 | #2 AND #4 | 152 | 39 | 9 | 20 |
| 10 | #3 AND #4 | 158 | 38 | 10 |  |
| 11 | #1 AND #2 AND #3 | 949 | 1,417 | 621 |  |
| 12 | #1 AND #2 AND #4 | 48 | 16 | 3 | 6 |
| 13 | #1 AND #3 AND #4 | 48 | 15 | 8 |  |
| 14 | #2 AND #3 AND #4 | 109 | 24 | 3 |  |
| 15 | #1 AND #2 AND #3 AND #4 Filter: Randomized Controlled Trail | 30 | 13 | 2 | 0 |

**The search strategy for other sources (Hand searching)**

Two reviewers identified additional references by checking the reference lists of identified articles.
