# Supplementary material for: Effects of different types and frequencies of early rehabilitation on ventilator weaning among patients in intensive care units: A systematic review and meta-analysis
Source: PLoS One. 2023 Apr 24;18(4):e0284923. doi: 10.1371/journal.pone.0284923 (PMC10124886; doi:10.1371/journal.pone.0284923)
Supplement: S1 Table — (DOCX) [file pone.0284923.s003.docx]

S2 Table. Characteristics of recruited studies

| Study  (Country) | Setting | Medical status  and sedation condition | Age  Mean (SD) or  Median (IQR) | Number of subjects | Intervention group | Control group | Outcome  measure | Study findings  Mean ± SD or  Median (IQR) |
| --- | --- | --- | --- | --- | --- | --- | --- | --- |
| Brummel, 2013 (America) | MICU  SICU | **Critical illness**   - Respiratory diseases - Cardiac diseases - Medical conditions - Surgical conditions   **Sedation condition:**  Controlling patients to keep RASS -1 ~ +1 | **Intervention 1:**  60 (51–69)  **Intervention 2:** 62 (48–67)  **Control:**  62 (54–69) | **Intervention 1:** 22  **Intervention 2:** 43  **Control:** 22 | **Multi-component**  **Intervention 1 (PM)**  1 time/day; daily  **Intervention 2 (PM + CT)**   - PM: 1 time/day; daily - CT: 20 mins/time; 2 times/day; daily | **PM**  1–2 times/week | Ventilator-free day during the first 30 days of hospital stay | NS  Intervention1:  27.1 (1.7-28.7)  Intervention2:  25.3 (0.0-28.9)  Control:  27.4 (0.0-29.2) |
| Cader, 2012  (Brazil) | ICU | **Bed-ridden elderly**   - Respiratory diseases - Surgical conditions - Trauma   **Sedation condition:**  No mention | **Intervention:**  82 (4)  **Control:**  81 (6) | **Intervention:** 14  **Control:**  14 | **CPT**   - Breathing exercise: 5 mins/time; 2 times/day; daily - PT (Positioning + ROM exercise): 15 min | **CPT**  PT (Positioning + ROM exercise): 15 min | 1. Extubation rate 2. Ventilator duration | 1. NS   Intervention: 11/14  Control: 8/14   1. NS   Intervention: 3.64 (1.50)  Control:  5.36 (1.87) |
| Chang, 2011  (Taiwan) | SICU | **Critical illness**   - Surgical conditions - Trauma   **Sedation condition:**  No mention | **Intervention:**  65.3 (13.1)  **Control:**  66.9 (17.0) | **Intervention:** 18  **Control:** 16 | **CPT**   - PT with sitting position: 30-120 mins/time; 1 time/day; $\geq$ 3 times/week | **Usual care**  Usual care in supine or semirecumbent position | 1. Ventilator duration 2. Extubation rate 3. Re-intubation rate | 1. NS   Intervention: 6.4 (4.0)  Control:  6.9 (5.3)   1. NS   Intervention:  15/18  Control: 13/16   1. NS   Intervention: 2/18  Control: 1/16 |
| Dantas, 2012  (Brazil) | ICU | **Critical illness**   - Respiratory diseases - Cardiac diseases - Surgical conditions - Medical conditions   **Sedation condition:**  Starting interventions after sedatives were withdrawn | **Intervention:** 59.07 (15.22)  **Control:**  50.43 (20.45) | **Intervention:** 14  **Control:** 14 | **Multi-component (CPT + EPT)**   - EM   Passive: 4 limbs 10 times/set  Sitting$\geq$ 20 mins; 2 times/day; 7 times/week   - Cycling - Balance training | **CPT**  5 times/week | Ventilator duration | NS |
| Denehy, 2013  (Australia) | ICU | **Critical illness**   - Respiratory diseases - Cardiac diseases - Surgical conditions   **Sedation condition:**  Controlling patients to keep RASS -1 ~ +1 | **Intervention:** 61.4 (15.9)  **Control:**  60.1 (15.8) | **Intervention:** 74  **Control:** 76 | **Multi-component (EPT + PM)**  MV: 15mins/day  Weaned: 2×15mins/day   - PM - Muscle strengthening   Target Modified Borg Scale score 3 to 5 | **Multi-component**  **(CPT + PM)**   - Respiratory care: daily - PM: daily | 1. Ventilator duration 2. Ventilator rate at day 5 | 1. NS   Intervention: 105.0 (52.0-216.5) hrs  Control:  98.0 (47.5-160.5) hrs   1. NS   Intervention: 41/74  Control: 42/76 |
| Dong, 2014  (China) | ICU | **Critical illness**   - Respiratory diseases - Medical conditions   **Sedation condition:**  Sedation interruption for 2 hours before training | **Intervention:** 55.3 (16.1)  **Control:**  55.5 (16.2) | **Intervention:** 30  **Control:** 30 | **PM**  2 times/day | **Medical Tx** | Ventilator duration | S  Intervention:  5.6 (2.1)  Control:  7.3 (2.8) |
| Dong, 2016  (China) | SICU | **CABG**  **Sedation condition:**  Sedation interruption for 2 hours before training | **Intervention:** 62.6 (12.8)  **Control:**  60.2 (15.1) | **Intervention:** 53  **Control:** 53 | **PM**  2 times/day | **Medical Tx** | Ventilator duration | S  Intervention:  8.1 (3.3)  Control:  13.9 (4.1) |
| Dong, 2021  (China) | ICU | **Critical illness**   - Surgical conditions   **Sedation condition:**  No mention | **Intervention:** 59.05 (17.61)  **Control:**  64.44 (14.72) | **Intervention:** 39  **Control:** 41 | **PM**  1 time/day; daily | **Medical Tx** | 1. Ventilator duration (Tracheotomy or Endotracheal intubation) 2. Intubation duration (Only endotracheal intubation) | 1. S   Intervention:  7.49 (2.59)  Control:  9.41 (5.32)   1. S   Intervention:  8.31 (2.80)  Control:  10.37 (5.32) |
| Fischer, 2016  (Austria) | SICU | **Patients s/p cardiothoracic surgery**   - Valve reconstruction or replacement - CABG   **Sedation condition:**  Providing average dosage of sedatives among patients | **Intervention:** 63.3 (15.5)  **Control:**  69.7 (13.1) | **Intervention:** 27  **Control:** 27 | **NMES**  $\geq$30 min/time; 2 times/day (<14 days)  Highest tolerable intensity just below the pain threshold; Biphasic rectangular pulses of 0.4 ms pulse duration at 66 Hz  The duty cycle was 3.5 s on and 4.5 s off, ramping up and down were both set at 0.5 s Dual Snap (5 × 10 cm and 5 × 5 cm) electrodes | **Usual care**  Sham NMES: same frequency as intervention group but no electrical stimulation | Ventilator duration | NS  Intervention:  2 (1-7)  Control:  2 (1-15) |
| Kho, 2015  (America) | ICU | **Critical illness**   - Respiratory diseases - Medical conditions   **Sedation condition:**  Average score of RASS   - Intervention: -1.8 - Control: -1.1   NS between groups | **Intervention:** 54 (16)  **Control:**  56 (18) | **Intervention:** 18→16  **Control:** 18 | **Multi-component (PM +NMES)**   - NMES: 60 mins/day **Quadriceps**: pulse duration was 400 μs with on-time of 5 s and off-time of 10 s   **TA and Gastrocnemius**: pulse duration was 250 μs, on-time 5 s and off-time 5 s   - PM: NI | **PM**   - PM: NI - Sham NMES: Same frequency as intervention group but no electrical stimulation | Ventilator duration | NS  Intervention:  20 (18)  Control:  16 (15) |
| Kurtoğlu, 2015  (Turkey) | ICU | **COPD**  **Sedation condition:**  No mention | **Intervention:** 66.06 (13.86)  **Control:**  69.93 (11.0) | **Intervention:** 15  **Control:** 15 | **Multi-component (CPT + NMES)**   - AROM exercise: according to patients’ tolerance in early stage, 10min/day on following days for 4weeks - NMES: 20 mins/day for 10 days   Amplitude was switched between 20 and 25 mA, and wave frequency was 50 Hz Biphasic symmetrical waves with 6 s duration of contraction, 1.5 s of increase, and 0.75 s of decrease were used | **CPT**  AROM exercise: same frequency as intervention group | Ventilator duration | NS  Intervention: 14.60 (10.97) Control:  18.13 (11.08) |
| McCaughey, 2019  (Australia) | ICU | **Critical illness**   - Respiratory diseases - Neurological diseases - Surgical conditions - Trauma   **Sedation condition:**  No mention | **Intervention:** 56.5 (38.0-75.0)  **Control:** 61.0 (43.8-78.25) | **Intervention:** 10  **Control:** 10 | **NMES**  30 mins; 2 times/day; 5 days/week (including first 5 days consecutively), until discharged from ICU  median 60mA [range 50–65mA], with frequency of 30Hz and pulsewidth of 350 μs | **NMES**  Same frequency as intervention group  10mA (sensory input without muscle contraction) | Extubation rate | NS  Intervention: 8/10  Control:  5/10 |
| Morris, 2016  (America) | MICU | **Critical illness**   - Respiratory diseases - Medical conditions   **Sedation condition:**  Percentage of duration with RASS score of 4 or 5 in ICU   - Intervention: 14.6% - Control: 14.3%   NS between groups | **Intervention:** 55 (17)  **Control:**  58 **(**14) | **Intervention:** 150  **Control:** 150 | **Multi-component (CPT + EPT)**  1 times/day; daily until hospital discharge   - PROM exercise: 5 repetitions for each upper and lower extremity joint - PT - Muscle strengthening: 8 reps/set; 3 sets/day (start using tan colored Thera-Bands which exert 1.1 pounds of force at 100% elongation) | **Usual care**  Receive routine care (PT could be ordered) and PT evaluations | Ventilator-free days during the first 28 days of hospital stay | NS  Intervention:  24 (19-26)  Control:  24 (20-26) |
| Moss, 2016  (America) | ICU | **Respiratory Failure**   - Respiratory diseases - Surgical conditions   **Sedation condition:**  Providing average dosage of sedatives among patients | **Intervention:** 56 (14)  **Control:**  49 (15) | **Intervention:** 59  **Control:** 61 | **Multi-component**  **(CPT + EPT +PM)**  30 mins/day, 7 days/week (up to 28 days)   - ROM exercise - Muscle strengthening: 6-8 reps/set; 3 sets/day - PM | **Multi-component**  **(CPT + PM)**  3 days/week (up to 28 days)   - ROM exercise: 10 mins - PM: 10 mins | Ventilator duration | NS  Intervention:  10 (7-18)  Control:  10 (7-19) |
| Nava, 1998  (Italy) | RICU | **COPD**  **Sedation condition:**  No mention | **Intervention:** 65 (6.90)  **Control:**  67 (9) | **Intervention:** 60  **Control:** 20 | **Multi-component**  **(CPT + EPT + PM)**  30-45 mins/time; 2 times/day   - Breathing exercise: Threshold device: at target pressure of 50% of MIP - Muscle strengthening - Cardiopulmonary exercise   Cycling: 20 mins  Climb flight of 25 stairs $\geq$5 times  Treadmill walking: 30 mins   - PM | **PM**  PM: 30-45 mins/time, 2 times/day  Ambulation: SaO2>88% | Extubation rate | Data is unclear |
| Patman, 2001  (Australia) | SICU | **Patients s/p cardiac surgery**   - Valve reconstruction or replacement - CABG   **Sedation condition:**  No mention | **Intervention:** 62.8 (12.2)  **Control:**  63.9 (14.4) | **Intervention:** 101  **Control:** 109 | **CPT**  Not specifically standardised or controlled   - Chest PT - ROM exercise | **Medical Tx**  PT after extubation | Ventilator duration | NS  Intervention: 13.0 (4.8) hrs  Control:  12.7 (4.7) hrs |
| Pattanshetty, 2011  (India) | MICU  SICU | **Critical illness**   - Respiratory diseases - Neurological diseases - Trauma - Cardiac diseases - Medical conditions - Surgical conditions   **Sedation condition:**  No mention | **Intervention:** 49.4 (16.13)  **Control:**  49.7 (16.21) | **Intervention:** 87  **Control:** 86 | **CPT**  2 times/day until patients weaned from ventilator   - Multimodality chest PT - Suctioning | **Usual care**  2 times/day   - MH - Suctioning | 1. Ventilator duration 2. Weaning Duration 3. Extubation rate | 1. NS. Intervention:   7.6 (3.97)  Control:  6.8 (4.46)   1. NS   Intervention: 1.98 (0.73) Control:  2.10 (0.78)   1. S   Intervention: 58/87  Control: 28/86 |
| Pinkaew, 2020  (Thailand) | MICU | **Critical illness**  **Sedation condition:**  No mention | **Intervention1:** 75.32 (14.28)  **Intervention2:** 69.08 (16.96)  **Control:**  74.68 (15.23) | **Intervention 1**: 25  **Intervention 2**: 23  **Control**: 23 | **Multi-component**  **Intervention 1 (EPT)**   - EMEB: 30 mins; 10 times/set, 3 sets/day, 5 days/week   **Intervention 2 (CPT)**   - EM: 5 times/week - AROM exercise $\geq$20 mins - Transfer to chair $\geq$20 mins | **CPT**  5 times/week   - PROM exercise - AROM exercise - Breathing exercise | Ventilator duration | S  Intervention 1: 6.52 (4.40)  Intervention 2: 5.78 (2.74) Control:  12.82 (5.69) |
| Schweickert, 2009  (America) | MICU | **Critical illness**   - Respiratory diseases - Neurological diseases   **Sedation condition:**  Sedation interruption for 2 hours before training | **Intervention:** 57.7 (36.3–69.1)  **Control:** 54.4 (46.5–66.4) | **Intervention:** 49  **Control:** 55 | **PM**  1 time/day; daily | **PM**  Random (by primary care team order) | 1. Ventilator duration 2. Ventilator-free days during the first 28 days of hospital stay | 1. S   Intervention: 3.4 (2.3–7.3) Control:  6.1 (4.0–9.6)   1. NS   Intervention: 23.5 (7.4–25.6)  Control: 21.1 (0.0–23.8) |
| Staudinger, 2010  (Austria) | MICU | **Critical illness**   - Respiratory diseases - Neurological diseases - Cardiac diseases - Medical conditions   **Sedation condition:**  Controlling patients to keep Ramsay sedation scale 3~5 | **Intervention:** 59 (16)  **Control:**  60 (15) | **Intervention:** 75  **Control:** 75 | **CPT**   - Rotation therapy (change position continuously)   Continuously for 18 hrs/day; daily   - Percussion:12 reps/s for 1 min before suction | **Usual care**  Supine semirecumbent position between 30° and 45°  Change position every 2-4 hrs; daily | 1. Ventilator duration 2. Ventilator-free days during the first 28 days of hospital stay | 1. S   Intervention:  8 (5)  Control:  14 (23)   1. S   Intervention: 15 (10)  Control:  11 (10) |
| Templeton, 2007  (England) | ICU | **Critical illness**   - Respiratory diseases - Neurological diseases - Trauma - Cardiac diseases - Surgical conditions   **Sedation condition:**  No mention | **Intervention:** 57.7 (16.5)  **Control:**  58.2 (18.0) | **Intervention:** 87  **Control:** 85 | **CPT**  Assessment: 2 times/day; daily  frequency depends on PT’s evaluation   - PT (Positioning/Manual pulmonary hyperinflation/ General mobilization) - Airway clearance techniques (vibration, postural drainage with tracheal suctioning) | **CPT**  Assessment: 2 times/day; daily  frequency depends on PT’s evaluation   - General mobilization - Suctioning | 1. Ventilator duration 2. To become re-ventilated duration 3. Re-intubation rate | 1. S   Intervention: 15 (3–82)  Control:  11 (3–76)   1. NS   Intervention: 1(1-9)  Control: 3.5(1-6)   1. NS   Intervention: 11/87  Control: 12/85 |
| Waldauf, 2021  (Czech Republic) | ICU | **Critical illness**   - Respiratory diseases - Neurological diseases - Trauma - Cardiac diseases - Surgical conditions   **Sedation condition:**  Controlling patients to keep RASS -1~0 | **Intervention:** 59.9 (15.1)  **Control:**  62.3 (15.4) | **Intervention:** 75  **Control:** 75 | **Multi-component**  **(EPT + PM + NMES)**  2 times/day; 6 days/week   - Cycling in supine - PM - NMES: pulse width 250 μs, pulse frequency, 40 Hz 0–60 mA | **Multi-component**  **(CPT + PM)**  2 times/day; 6 days/week   - Chest PT - Positioning - ROM exercise - PM | Ventilator-free days during the first 28 days | NS  Intervention:  9.3 (1.4)  Control:  11.0 (1.4) |
| Wright, 2017  (England) | MICU  SICU | **Critical illness**  **Sedation condition:**  Controlling patients to keep RASS -1~ +1 | **Intervention:**  60 (16)  **Control:**  64 (16) | **Intervention:** 150  **Control:** 158 | **Multi-component**  **(EPT + PM)**  90mins; 5 times/week   - Muscle strengthening - PM | **Multi-component**  **(EPT + PM)**  30mins; 5 times/week   - Muscle strengthening - PM | Ventilator duration | NS  Intervention:  4 (3–7)  Control:  4 (3–6) |
| Yosef-Brauner, 2015  (Israel) | MICU  SICU | **ICUAW**  **Sedation condition:**  No mention | **Intervention:** 51.6 (18)  **Control:**  61.5 (12) | **Intervention**: 9  **Control**: 9 | **Multi-component**  **(CPT +PM)**  15 mins/time; 2 times/day   - Chest PT - PM | **Multi-component**  **(CPT +PM)**  15 mins/time; 1 time/day   - Chest PT - PM | Ventilator duration | NS  Intervention:  9 (5)  Control:  16.22 (2) |

AROM: Active Range Of Motion; CABG: Coronary Artery Bypass Graft; COPD: Chronic Obstruction Pulmonary Disease; CPT: Conventional Physical Therapy; CT: Cognitive Therapy; ICU: Intensive Care Unit; ICUAW: Intensive Care Unit-Acquired Weakness; IQR: InterQuartile Range; EM: Early mobilization; EMEB: Early Mobilization with Elastic Band; EPT: Exercise-based Physical therapy; MH: Manual Hyperinflation; MICU: Medical Intensive Care Unit; MIP: Maximal Inspiratory Pressure; NI: No Information; NMES: Neuromuscular Electrical Stimulation; NS: No Significant; PROM: Passive Range Of Motion; PM: Progressive Mobility; PT: Physical Therapy; RASS: Richmond Agitation-Sedation Scale; RCC: Respiratory Care Center; RICU: Respiratory Intensive Care Unit; ROM: Range Of Motion; S: Significant; SD: Standard Deviation; SICU: Surgical Intensive Care Unit; SaO2: Arterial Oxyhemoglobin Saturation; s/p: status post; TA: Tibialis Anterior; Tx: Treatment
