# Supplementary material for: Effects of different types and frequencies of early rehabilitation on ventilator weaning among patients in intensive care units: A systematic review and meta-analysis
Source: PLoS One. 2023 Apr 24;18(4):e0284923. doi: 10.1371/journal.pone.0284923 (PMC10124886; doi:10.1371/journal.pone.0284923)
Supplement: Attachment — Submitted filename: Response to Reviewers.docx [file pone.0284923.s005.docx]

**Journal Requirements:**

**Please review your reference list to ensure that it is complete and correct. If you have cited papers that have been retracted, please include the rationale for doing so in the manuscript text, or remove these references and replace them with relevant current references. Any changes to the reference list should be mentioned in the rebuttal letter that accompanies your revised manuscript. If you need to cite a retracted article, indicate the article’s retracted status in the References list and also include a citation and full reference for the retraction notice.**

**Reply:**

We confirmed that our reference list was correct and complete without any retracted articles.

**Reviewers' comments:**

**1. If the authors have adequately addressed your comments raised in a previous round of review and you feel that this manuscript is now acceptable for publication, you may indicate that here to bypass the “Comments to the Author” section, enter your conflict of interest statement in the “Confidential to Editor” section, and submit your "Accept" recommendation.**

**Reviewer #1: (No Response)**

**Reviewer #2: All comments have been addressed**

**Reply:** Thank you for your thoughtful suggestions and insights, which have enriched the manuscript and produced a better and more balanced account of the research.

**2. Is the manuscript technically sound, and do the data support the conclusions?**

**Reviewer #1: Yes**

**Reviewer #2: Yes**

**Reply:** Thank you for your comment.

**3. Has the statistical analysis been performed appropriately and rigorously?**

**Reviewer #1: Yes**

**Reviewer #2: Yes**

**Reply:** Thank you for your comment.

**4. Have the authors made all data underlying the findings in their manuscript fully available?**

**Reviewer #1: Yes**

**Reviewer #2: Yes**

**Reply:** Thank you for your comment.

**5. Is the manuscript presented in an intelligible fashion and written in standard English?**

**Reviewer #1: Yes**

**Reviewer #2: Yes**

**Reply:** Thank you for your comment.

**6. Review Comments to the Author**

**Reviewer #1: (No Response)**

**Reviewer #2:**

**Thank you for responding to all concerns and for a great job. just a few minor comments**

**Line 227, page 68 of the pdf document….Review Manager or manager? 5.4 capitalize the letter "m" or not?
Line 271. Table 1. Europe, USA, South America are not Races or racial categories. They are geographical.**

**Lastly, I think that the native English speaker/writer did a wonderful job. However, there are still a few sections or segments or fragments that could use improvement.**

**Reply:**

Thanks for your comment. We revised **Review manager** to **Review Manager** and revised **Race** to **Geographical region** in the Table 1. In order to ensure the readability or logical flow of study, the manuscript was proofread and edited by native speaker again. All changes were highlighted in the file “Revised Manuscript with Track Changes”.
